# A metasurface-based light-to-microwave transmitter for hybrid wireless communications

**DOI:** 10.1038/s41377-022-00817-5

**Published:** 2022-05-06

**Authors:** Xin Ge Zhang, Ya Lun Sun, Bingcheng Zhu, Wei Xiang Jiang, Qian Yu, Han Wei Tian, Cheng-Wei Qiu, Zaichen Zhang, Tie Jun Cui

**Affiliations:** 1 State Key Laboratory of Millimeter Waves, School of Information Science and Engineering, Southeast University, 210096 Nanjing, China; 2 National Mobile Communications Research Laboratory, School of Information Science and Engineering, Southeast University, 210096 Nanjing, China; 3 Frontiers Science Center for Mobile Information Communication and Security, Southeast University, 210096 Nanjing, China; 4 Purple Mountain Laboratories, 211111 Nanjing, China; 5 Department of Electrical and Computer Engineering, National University of Singapore, Singapore, 117583 Singapore

**Keywords:** Electronics, photonics and device physics, Metamaterials, Microwave photonics

## Abstract

Signal conversion plays an important role in many applications such as communication, sensing, and imaging. Realizing signal conversion between optical and microwave frequencies is a crucial step to construct hybrid communication systems that combine both optical and microwave wireless technologies to achieve better features, which are highly desirable in the future wireless communications. However, such a signal conversion process typically requires a complicated relay to perform multiple operations, which will consume additional hardware/time/energy resources. Here, we report a light-to-microwave transmitter based on the time-varying and programmable metasurface integrated with a high-speed photoelectric detection circuit into a hybrid. Such a transmitter can convert a light intensity signal to two microwave binary frequency shift keying signals by using the dispersion characteristics of the metasurface to implement the frequency division multiplexing. To illustrate the metasurface-based transmitter, a hybrid wireless communication system that allows dual-channel data transmissions in a light-to-microwave link is demonstrated, and the experimental results show that two different videos can be transmitted and received simultaneously and independently. Our metasurface-enabled signal conversion solution may enrich the functionalities of metasurfaces, and could also stimulate new information-oriented applications.

## Abstract (web summary)

A metasurface-based light-to-microwave transmitter, which can convert a light intensity signal to two microwave signals directly, was proposed to achieve the dual-channel data transmissions in light-to-microwave hybrid wireless communications.

## Graphical abstract

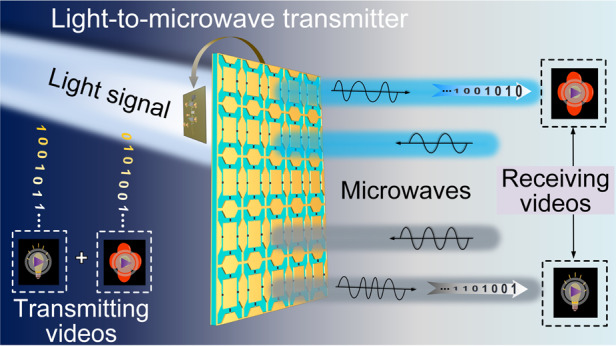

## Introduction

Signal conversion between optical and radio frequency (RF) domains is the basis and key to realize hybrid systems employing both optical and RF communication technologies^1–5^. The hybrid optical and RF communication systems can release the limitations of the individual systems and provide positive features of them, which is identified as a promising way to support multi-domain integrated and full-spectrum networks for future sixth generation (6G) wireless communications^6–10^. So far, the mixed communications are realized typically through the cooperative relaying systems where the received optical signals (or RF signals) are firstly amplified and converted to baseband before being down-converted to the RF (or up-converted to optical) domains. This solution needs a large number of optical components, RF devices, and multiple process steps that eventually make the hybrid systems with high cost and high complexity. Recently, plasmonic-enabled schemes for direct conversions of millimeter and terahertz waves to the optical signals have been implemented, which lead to a significant leap forward toward the low-complexity and cost-efficiency wireless-to-fiber communication systems^2,3^. In contrast to that, the device that can encode directly a light signal onto a microwave carrier for the optical-to-RF hybrid wireless communication has not yet been demonstrated.

Metasurfaces are two-dimensional (2D) artificial structures with precisely engineered elements in subwavelength scales, which have unfolded many remarkable approaches to manipulate the electromagnetic (EM) waves and overcome several metamaterial challenges of bulky, lossy and fabrication, enabling a broad range of applications^11–26^. As an emerging technology, programmable metasurfaces allow real-time EM manipulations and information processing, which have shown great potentials to construct RF-chain-free transmitters and new paradigms of 6G intelligent and programmable wireless environment^27–33^. However, the mostly used electrical control method to date limits the programmable metasurfaces to be only considered in a single domain and thus blocks their transition to advanced optoelectronic hybrid multi-physics platforms that can process the optical and RF signals simultaneously. We have previously reported the photodiode-based light-controllable microwave metasurfaces^13–15^, on which the EM functions can be programmed by visible light, but the scheme of just loading the photodiode array onto the metasurface is hard to use for wireless communication due to the slow switching speed.

In this article, we report a hybrid metasurface-based transmitter for direct signal conversion from visible lights to microwaves. Such the hybrid transmitter is implemented by an optically programmed time-varying metasurface, which is constructed via the heterogeneous integration of a high-speed and linear photoelectric detection circuit into a reflective programmable metasurface. The profile of the whole platform is around 0.04*λ* at 6.0 GHz. When receiving a modulated light signal, the metasurface platform is able to convert the light signal to the microwave domain directly without using down-conversion process to baseband. More importantly, one designed light signal can carry two independent original information that can be encoded onto two microwave carriers with different incidence frequencies by using the dispersion characteristic of the metasurface. Based on the light-to-microwave metasurface transmitter, we realize a dual-channel hybrid wireless communication system that can transmit simultaneously two different videos with the aid of frequency division multiplexing (FDM) scheme.

## Results

### Encoding a light signal onto two microwave carriers via metasurface

**Fig. 1:**
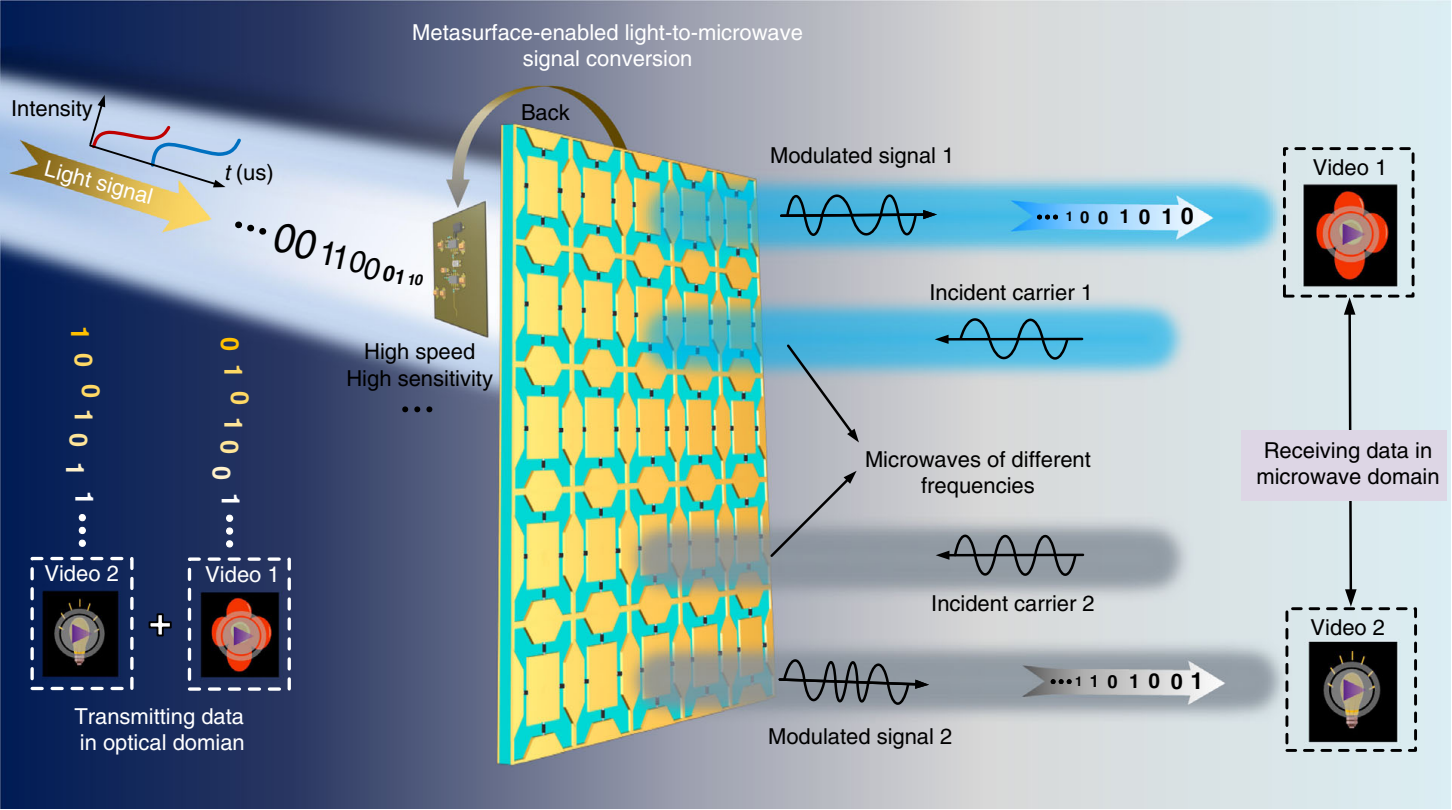
Schematic of direct light-to-microwave signal conversion using a hybrid time-varying metasurface platform. The metasurface platform is realized by integrating a high-speed and high-sensitivity photoelectric detection circuit into the back of a programmable microwave metasurface, where the reflection spectrum of metasurface can be manipulated by the light waveform in real time, thus providing a bridge to link the light signal and microwave signal. By further using the dispersion characteristics of the metasurface, one designed light intensity signal can be directly converted to two different BFSK signals for implementing FDM. Therefore, a light-to-microwave transmitter can be well realized. With this metasurface-enabled transmitter, a dual-channel light-to-microwave wireless link is built up, in which two different videos can be transmitted and received simultaneously and independently

Figure 1 presents the scheme for the direct conversion of a light signal to microwave signals by just using a single planar hybrid time-varying metasurface platform, without any additional RF devices and optical components. The metasurface platform consists of a varactor-based dynamic wideband metasurface and a photoelectric detection circuit that is composed of a photodiode and two cascaded transimpedance amplifying circuits. With this hybrid integration strategy, the reflection phase of the metasurface can be modulated by light intensity at high speed. When the intensity of the illumination light changes periodically and rapidly in a special waveform, the metasurface will generate a certain reflected harmonic distribution based on the phase modulation under microwave incidence. Therefore, the digital information can be modulated on the waveforms of the light signal and then are mapped directly onto the spectral characteristics of the reflected microwaves, thus achieving the direct light-to-microwave signal conversion and transmission. In this case, the optically programmed time-varying metasurface can be used to construct the light-to-microwave transmitter. Moreover, we explore to use strong dispersion response of the designed metasurface to convert one light signal containing four different sets of modulated waveforms to two different binary frequency shift keying (BFSK) microwave signals. Therefore, two data (e.g., two different videos) can be converted and transmitted through the same metasurface aperture independently and simultaneously for implementing FDM, which improves greatly the information processing capability and efficiency of the metasurface platform.

To support the high bandwidth demand of wireless communications, the optical spectrum is considered as a promising complementary resource of RF, due to the good features of vast license-free spectrum, high security, high energy efficiency, and EM interference free. Compared to the widely used wireless transmission in microwave bands, the visible light communication (VLC) based on light waves is very suitable for many special application scenarios, such as the indoor communication, underwater communication, and data transfer in some EM sensitive environments including hospitals, gas stations, and underground mines. Because the microwave communication and VLC have complementary advantages in communication environments, seamless integration of VLC links into the existing RF wireless infrastructures is of crucial importance for full-spectrum wireless networks. We know that the VLC is a promising method for underwater communication due to relative long-distance communication and high speed, but it is easy to be blocked and disturbed in free space; conversely, microwave communication is widely used in free space, however, microwave has great loss under water and it is not conducive to long-distance transmission^7^. To overcome this limitation, the hybrid transmitter that allows light-to-microwave signal conversion and transmission is very necessary. For example, the light-to-microwave transmitter can be used to receive the modulated light signals from the underwater communication devices, and then transmit these data to the corresponding air receivers through the microwave link. Our proposed metasurface-enabled direct light-to-microwave signal conversion offers a practical and low-overhead solution to establish such a hybrid transmitter, where the complicated and distributed relay systems are no longer required. In addition, dual-channel data transmissions are achieved based on FDM for improving the capacity of the hybrid wireless network.

**Fig. 2:**
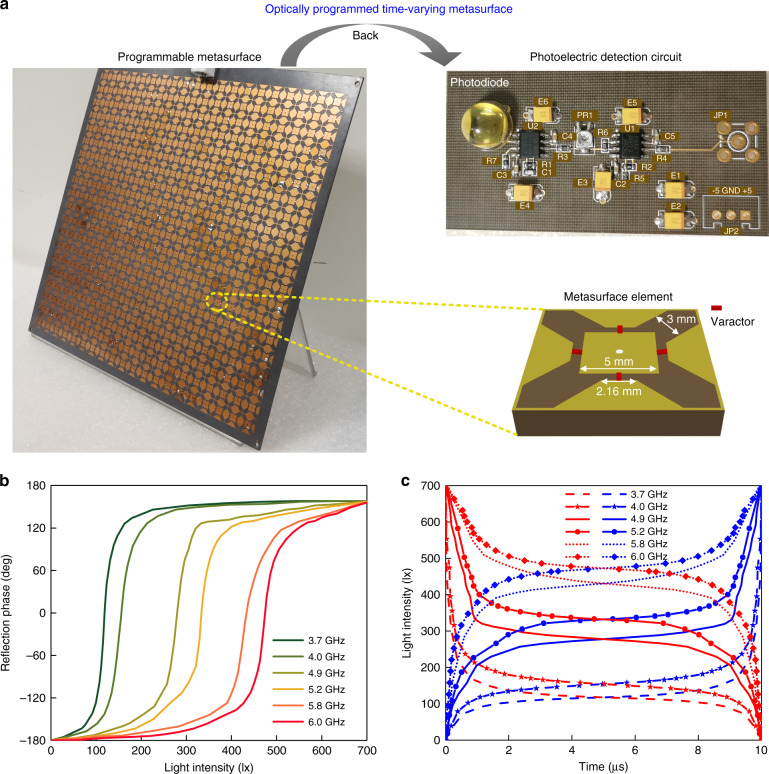
The optically programmed time-varying metasurface and its performance. **a** Fabricated hybrid metasurface platform, in which a photoelectric detection circuit is integrated on the back of the programmable metasurface. In the metasurface element, four identical varactors are loaded onto the four 0.7-mm-width gaps of the top metal pattern, respectively. **b** Measured reflection phases of the metasurface sample under the *x*-polarized incidence at several frequencies for different light intensities varying from 0 to 700 lx. **c** Up-modulation light waveforms (blue curves) and down-modulation light waveforms (red curves) with 100 kHz modulation frequency for efficiently generating the blue-shifted components and red-shifted components, respectively, at the corresponding frequencies

Time-varying metasurfaces with dynamic harmonic control capability have been demonstrated as an effective information delivery mechanism^34–38^. Different from the electrically controlled time-modulated metasurfaces, we design and fabricate an optically programmed time-varying metasurface (Fig. 2a), in which the reflection spectrum of metasurface can be controlled by visible light. Key to the multi-physics field platform is to integrate fully a microsecond level optoelectronic circuit into a microwave metasurface. The front of the hybrid metasurface sample is the arranged periodically dynamic metasurface elements, each of which has a 90° rotationally symmetric metal pattern printed on a polytetrafluoroethylene glass cloth copper clad laminate (F4B) substrate with a dielectric constant of 2.65 and a loss tangent of 0.001. Four identical varactors are symmetrically embedded onto the metal structure for realizing polarization-insensitive and broadband features. Since the resonant frequency of the element is related to the capacitance value, the element bandwidth mainly depends on the capacitance variation range of the used varactor. Here, we adopt the “MAVR-000120-14110P” varactor with a high capacitance ratio of 8.2 (varying from 1.15 to 0.14 pF) to realize the wideband tunability. The back of the metasurface sample is a photoelectric detection circuit, in which the cascaded amplifying circuits are designed to enable the photoelectric detection circuit to generate a large voltage variation range linearly and rapidly. Compared with the series photodiode array in our previous work^13^, the switching frequency of the photoelectric detection circuit is about 10000 times higher and can reach 2 MHz. In such a case, when receiving the visible light with different intensities, the photoelectric detection circuit will convert them to the corresponding voltages for tuning the loaded varactors, and then changes the reflection phase of the incident microwaves in real time. See “Materials and Methods” for more details on circuit principle as well as sample design and fabrication.

Spectrum manipulation depends on the accurate temporal control of the reflection phase. Thus, we firstly test the reflection phases of the metasurface sample at several frequencies for different light intensities, and the measured results are plotted in Fig. 2b. It is clear that when the illumination intensity increases gradually from 0 to ~700 lx, the reflection phase will change accordingly from −180° to around 160°, offering about 340° phase excursions at all test frequencies. We have proved that when the reflection phase changes linearly in one time period *T* = 1/*f*_t_, the time-varying metasurface will generate a frequency shift *f*_t_ upon reflection^39^. We here use the visible light to drive the metasurface for achieving the high-efficiency frequency manipulation. The required up- and down-modulation optical waveforms are derived from the measured reflection phase curves, which allow the reflection phase to change linearly with time (Fig. 2c). Controlled by these up- and down-modulation waveforms, the metasurface sample can produce effectively blue- and red-shifts, respectively, corresponding to the frequency shift keying (FSK) in digital modulation formats (see Supplementary Note 1 for more details). Hence, a light waveform signal can modulate directly the reflection frequency of the metasurface, indicating that the hybrid metasurface platform provides an interface to link the light signal with the microwave FSK signal in real time.

The measured phase curves show a strong dispersion response of the metasurface, as discussed in Supplementary Note 2. Due to the strong dispersion, the required modulation waveforms for efficient frequency conversion at different operating frequencies are obviously different, as shown in Fig. 2c. See Supplementary Note 3 for details on the characteristic of light waveforms. Because the frequency conversion efficiency of the time-varying metasurface highly relies on the control waveform, for a fixed light waveform, the main reflection component can be at the harmonic (good conversion effect) or the fundamental frequency (poor conversion effect) under two microwave incidences with different frequencies. In this case, by employing the fundamental frequency and the frequency-shifted harmonic as two discrete frequencies to implement the BFSK modulation, one light waveform can represent two different digital symbols at these two incident frequencies. To achieve two BFSK signals simultaneously, the bandwidth selection of the two incident frequencies is crucial for implementing FDM. More details are provided in “Materials and methods”. As demonstrations, we adopt 5.2 and 4.9 GHz microwaves as the two single tone incident subcarriers. Without losing generality, we use the fundamental frequency and the blue-shifted frequency to represent the digital symbols ‘0’ and ‘1’, respectively.

**Fig. 3:**
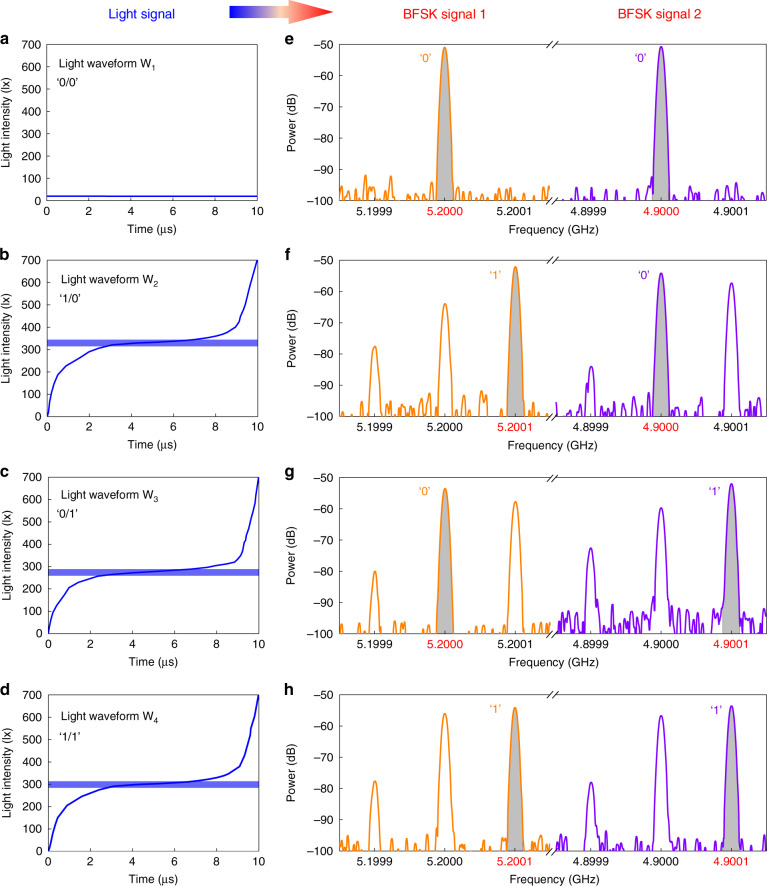
Process of converting one light signal to two BFSK signals. **a**–**d** Four different light waveforms of W_1_ (**a**), W_2_ (**b**), W_3_ (**c**), and W_4_ (**d**) in one light signal carry four sets of digital symbols. **e**–**h** Measured reflection spectral distributions of BFSK signal 1 (left column) and BFSK signal 2 (right column), in which the fundamental frequency and the blue-shifted frequency represent the digital symbols ‘0’ and ‘1’, respectively. When receiving the light waveforms W_1_-W_4_, the generated two BFSK signals are used to transmit ‘0/0’ (**e**), ‘1/0’ (**f**), ‘0/1’ (**g**), and ‘1/1’ (**h**)

The designed four light waveforms and the correspondingly measured spectral distributions of the metasurface at 5.2 and 4.9 GHz are plotted in Fig. 3, which illustrate the dual-channel signal conversion process. Two independent data streams are encoded onto the four sets of different light waveforms. The light waveform W_1_ is a direct-current control waveform (Fig. 3a), in which the light intensity is not changed with time and thus the frequencies of both reflected BFSK signals are at the original fundamental frequencies of 5.2 and 4.9 GHz (Fig. 3e). In this case, two digital symbols ‘0’ are transferred successfully from the light signal to the microwave domain. The light waveforms W_2_ and W_3_ are the up-modulation waveforms corresponding to 5.2 and 4.9 GHz, respectively (Fig. 3b, c). For light waveform W_2_, the main frequency component of BFSK signal 1 is at the blue-shifted frequency of 5.2001 GHz (related to bit ‘1’), but that of BFSK signal 2 is still at the fundamental frequency of 4.9 GHz (related to bit ‘0’) (Fig. 3f). Similarly, the light waveform W_3_ is used to encode the digital symbol ‘0’ onto BFSK signal 1 and digital symbol ‘1’ onto BFSK signal 2, as shown in Fig. 3g. The light waveform W_4_ in Fig. 3d is designed to encode the digital symbol ‘1’ onto two BFSK signals simultaneously, where the main frequency components are both blue-shifted frequencies (Fig. 3h). Hence, one light signal that contains the four sets of waveforms can be converted directly to two BFSK signals based on FDM. In this case, the hybrid light-to-microwave transmitter can be well realized by using the optically programmed time-varying metasurface.

### Implementation of a dual-channel hybrid communication system

**Fig. 4:**
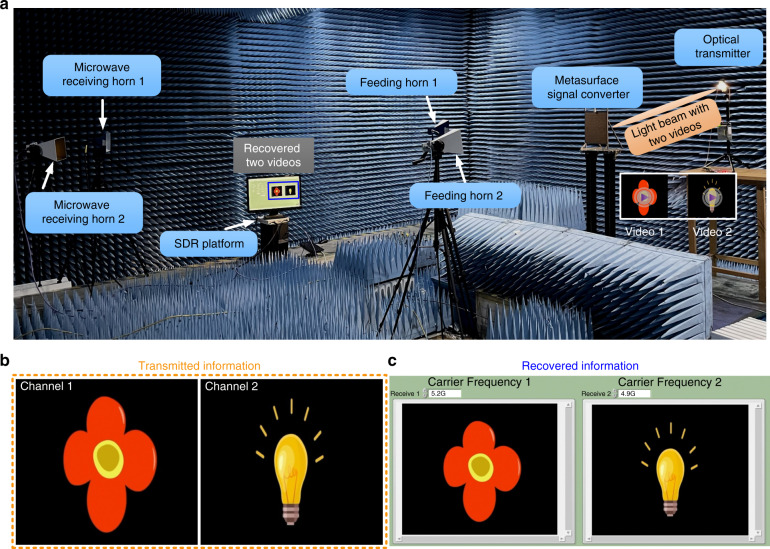
Experimental demonstration of the dual-channel hybrid communication system based on the metasurface-enabled light-to-microwave transmitter. **a** Photograph of the experiment setup. The hybrid communication system contains an optical transmitter, a metasurface signal converter, and a microwave receiver, in which two different videos can be transmitted and received simultaneously and independently in a light-to-microwave wireless link. **b**, **c** The original transmitted information of the flower blooming and the bulb glowing (**b**), and the corresponding recovered information (**c**), respectively

To further verify the proposed scheme of direct light-to-microwave signal conversion and show its potential applications, we build a dual-channel hybrid wireless communication system based on the hybrid metasurface transmitter, as shown in Fig. 4a. The demonstrated hybrid communication system mainly consists of three parts: an optical transmitter, a metasurface signal converter, and a microwave receiver. The optical transmitter contains a light modulation and driving module and a light-emitting diode (LED) light source, which is designed to generate the light signals with the modulated information. In the optical domain, the metasurface-enabled light-to-microwave signal converter acts as an optical receiver for capturing the encoded light signals, while it works as a RF transmitter in the microwave communication link. Two horn antennas in the transmitter are used to provide two incident carriers with different frequencies simultaneously. The microwave receiver mainly contains two receiving horn antennas and a software-defined radio (SDR) platform (NI USRP-2954) connected to a post-processing computer. We remark that the hybrid communication system can implement a light-to-microwave wireless link where complicated relay systems are no longer required. See “Materials and methods” for more details on the system prototype.

It is important to note that unlike the traditional wireless transmitters, the metasurface-based transmitter is able to modulate directly the baseband signal onto the air-fed carrier waves. In this case, the real-time programmable capability is very critical. For the traditional RF emission sources, such as horn antennas and microstrip antennas, the programmability is hard to achieve, especially for multi-element array. As a typical reconfigurable technology, the phased array antenna has been proposed and realized to control EM waves dynamically. However, the conventional phased array antennas are expensive, and their complex feeding structures and transmitter/receiver modules will affect the performance of antennas. Compared with the phased array antennas, the programmable metasurfaces usually have advantages of simple design and low cost. Moreover, the programmable metasurface can be designed elaborately for achieving the wideband feature to realize the FDM.

To show the communication capability of our constructed light-to-microwave hybrid wireless system, as an example, we demonstrate a real-time dual-channel video transmission based on the system. In experiments, two different videos (one is the flower blooming and the other is the bulb glowing, Fig. 4b) were firstly converted into two sets of bit streams that are then encoded onto the light signals with four sets of waveforms W_1_–W_4_ through the optical transmitter. Two subcarriers at the frequencies of 5.2 and 4.9 GHz are emitted from the feeding horn antenna 1 and horn antenna 2, respectively, for exciting the metasurface simultaneously. The light signal with two modulation information (videos 1 and 2) are converted directly to BFSK signals 1 and 2, respectively, by using the above-discussed signal conversion process. These two reflected BFSK signals are then received individually by the two horns and demodulated into the digital baseband signals via the SDR receiver to recover the two videos independently and simultaneously, as shown in Fig. 4c (see “Materials and methods” for detailed workflow of the system prototype). The entire transmission process is given in Supplementary Movie 1 and is described briefly in Supplementary Note 4. The transmission rate of the hybrid communication system is 100 kbps. The results validate that the two videos can be transmitted successfully from the optical transmitting terminal to the microwave receiving terminal by the metasurface-enabled light-to-microwave transmitter.

## Discussion

It should be noted that by designing the higher frequency metasurfaces, the proposed hybrid integration strategy could be extended to achieve other types of signal conversion platforms, such as the light-to-terahertz transmitter. Recently, several remarkable tunable components, such as complementary metal-oxide-semiconductor (CMOS)-based chip tile^40^ and microelectromechanical systems (MEMS)^41^ have been demonstrated for realizing programmable terahertz metasurfaces. By combining these programmable terahertz metasurfaces with appropriate design of photoelectric detection circuit, it is possible to extend our concept of hybrid communication system to higher frequencies, which will help to develop more multi-physics metasurfaces, significantly expanding the potential applications of the metasurfaces. In fact, except for communication application, metasurfaces have also shown many other interesting functions, such as multichannel 3D meta-holography^42^, off-axis multi-color imaging^43^, dynamic full-color digital holographic display^44^ and metasurface-based quantum photonics^45^.

Although the proposed joint control scheme of the photodiode-based circuit and varactor is very effective and robust^13–15,46^, it will add an additional photoelectric conversion step. To achieve the all-optical tuning, one promising solution is to integrate photodiode or photosensitive material such as silicon^47,48^ into the metasurface element directly. In this case, the EM response of metasurface can be controlled by changing the characteristics of photosensitive components with light illumination, without involving any electrosensitive diodes.

In summary, we have demonstrated a metasurface-based transmitter that enables direct signal conversion from the visible light to microwaves for VLC/RF hybrid communication applications. The low-profile hybrid metasurface platform is realized by integrating a photoelectric detection circuit into a time-varying microwave metasurface. The experimental results verified that the reflection frequency of the metasurface platform can be controlled by the light intensity waveform in real time, thus achieving the FSK modulation. We have also presented an actual scheme of simultaneously converting one light signal to two BFSK signals by utilizing the dispersion characteristics of the metasurface. To demonstrate our approach, we constructed and tested a dual-channel hybrid wireless communication system, in which two different videos can be transferred from the optical transmitter to microwave receiving terminal simultaneously and independently based on the FDM technology. Because the light-to-microwave signal conversion process can be completed fully on a single platform, our metasurface-enabled transmitter shows extraordinary potentials to implement low-cost and low-complexity hybrid communication systems, which are fundamentally important to multi-domain integrated 6G wireless communications^49,50^.

## Materials and methods

### Working principle of the photoelectric detection circuit

The simplified diagram of the designed photoelectric detection circuit is presented in Fig. S1. Under light illumination, the photodiode will produce a weak photocurrent *I*_pd_, which can be converted into voltage by the transimpedance amplifying circuit and the output voltage is *U*_2_ = *I*_pd_ × *R*_1_. Then, the voltage *U*_2_ will be further amplified by the post amplifying circuit, and the final generated driving voltage is *U*_1_ = −*U*_2_ × *R*_2_/*R*_3_. In this case, the maximum output voltage of the photoelectric detection circuit can be controlled by tuning the resistance values. Moreover, except for the amplification capability, the cascaded amplifying circuit also provides a low-resistance current loop for the photodiode. Therefore, the photodiode is able to complete the charge-discharge process quickly, resulting in a high switching speed. As a comparison, we have measured the response speeds of the designed photoelectric detection circuit and the series photodiode array in our previous work^13^. In experiments, we use the time-varying light with the periodic square wave frequency to illuminate these two different photodiode circuits, and the measured output voltage waveforms are shown in Fig. S2a, b, respectively. It is obvious that the switching frequency of the photoelectric detection circuit is able to reach 2 MHz, but that of the photodiode array is only about 200 Hz. The reason is that the previous photodiode array has no current loop and thus the charges are difficult to release from the photodiodes, which can also be seen from the falling edge of its output square wave.

### Sample design and fabrication

The size of metasurface element is 12 mm × 12 mm and the height of the grounded F4B dielectric is optimally set as 2 mm. The other geometric parameters of the element are shown in Fig. 2a. The metasurface element is identical to that in our previous work^39^, but is controlled by light here. To achieve a high reflectivity and a large phase difference, the frequency range of the element is preferably 4.0 to 6.3 GHz. More details on the analysis and results of the element can be found in ref. ^39^. The preamplifier connected to the “S6968” photodiode is a low-noise “OPA657U” amplifier, which allows the generated weak photocurrent to be detected accurately. The post-amplifier connected to the metasurface is an “AD8065ARZ” amplifier with strong driving capability. With this configuration, the high-speed photoelectric detection circuit is linear and can generate a maximum voltage of ~10 V under light illumination. The fabricated sample contains 20 × 20 elements and all the elements are driven uniformly by the connected photoelectric detection circuit. Because the bare dielectric area around the metasurface elements is without a metal ground and this area is very small compared with the periodic element array, it has almost no effect on the reflection performance of the metasurface. The measured reflection efficiencies of the metasurface sample at two working frequencies are 67.2% and 66.4%, respectively. The loss of the metasurface mainly comes from the equivalent resistance of the used varactors. To improve the energy efficiency, we can select carefully the varactor with a smaller equivalent resistance. Recently, a beneficial strategy has been proposed to design and realize the self-filtering metasurface with pure modulated wave^24^, which is able to improve the transfer efficiency by 83% compared with the traditional approach.

### Frequency interval of two subcarriers in FDM

The measured spectral distributions of the time-varying metasurface (Fig. S3a–c) show that the reflected frequency can be controlled by light signal in real time. The two symmetrically distributed blue-shifted and red-shifted components can be used naturally to provide a pair of discrete frequencies in BFSK. But in this manner, one light signal can only be converted to one BSFK signal. To generate two BSFK signals, the two incident subcarriers should be selected first. We remark that the bandwidth of the subcarriers is very important. If the bandwidth is too narrow, a light waveform will generate the similar frequency modulation effect at the two frequencies and thus it cannot represent two different digital information simultaneously. If the frequency interval is too large, it is difficult to design a light waveform to produce high-efficiency blue shifts at both frequencies. According to multiple measurement results, we find that the dual-channel signal conversion can be realized via the FDM scheme when the bandwidth of two subcarriers is ~300 MHz.

### Details on system prototype

The hybrid communication system was built in a microwave chamber. The light modulation and driving module were implemented by an FPGA and a digital-to-analog converter (DAC). To generate the required light waveform accurately, a linear focusing LED light source was designed and fabricated. In the experiments, the distance from the light source to the back of the metasurface sample was 2.0 m. In this case, the generated light beam with modulation waveforms can be well focused onto the photoelectric detection circuit. The feeding horn antennas 1 and 2 were connected to an Agilent N5230C vector network analyzer and a Keysight E8267D signal generator to emit the monochromatic waves with frequencies of 5.2 and 4.9 GHz, respectively. The height of the horn antenna 1 is 136 mm, and the length and width of the horn aperture are 150 and 114 mm, respectively. The corresponding physical dimensions of the horn antenna 2 are 260, 170 and 120 mm, respectively. Both feeding horns are 2.4 m away from the front of the metasurface sample. Because the metasurface requires the primary feed antenna, it will increase the volume of the whole communication system. To overcome this limitation, one promising solution is to develop the direct radiation-type metasurfaces. Recently, two low-profile radiation-type metasurfaces have been verified experimentally for controlling EM waves flexibly^25,26^, without any air-feed antennas. The receiving horns 1 and 2 were fixed ~7.0 m away from the metasurface sample to receive the reflected BFSK signals 1 and 2, respectively. Due to the property of the FDM scheme, the interference between the two nearby receiving horn antennas can be well eliminated.

### Workflow of the hybrid communication system

The light-to-microwave hybrid communication is performed in three stages. Firstly, each pixel of videos 1 and 2 is represented by a 32-bit binary sequence, and thus the two videos can be converted into two sets of bit streams (such as ‘01010…’ and ‘10010…’). The two sets of bit streams stored in FPGA are read bit by bit at the same time, combining into a set of 2-bit binary digits. The four states of the 2-bit binary digits are translated into different control signals through the FPGA and DAC modules for driving the light source to produce the corresponding light waveforms. Fig. S4a schematically shows the above mapping process. Secondly, when receiving the light signal, the metasurface platform will generate four sets of spectral distributions for implementing the dual-channel BFSK modulations under two subcarriers incidences. Finally, the two reflected BFSK signals are received by the RF receiver for processing. For BFSK demodulations, the time domain signal is first transformed into the frequency domain by using fast Fourier transform. Then in each channel the two frequency components of the fundamental frequency (*f*_L_) and blue-shifted frequency (*f*_R_) are compared with each other. If the power of the fundamental frequency is larger than that of the blue-shifted frequency, the received digital symbol is determined to be ‘0’; on the contrary, the received digital symbol is determined to be ‘1’, as conceptually shown in Fig. S4b. It should be noted that from the measured spectral distributions in Fig. 3, we clearly see that the energy differences between the fundamental frequency and blue-shifted frequency are larger than 3 dB for all cases, which is large enough for the BFSK demodulations. After demodulation, two sets of bit streams are obtained and then the original videos are recovered using the post-processing programs. The complete workflow of the hybrid communication system is depicted in Fig. S4c.

## Supplementary information

Supplementary information for A metasurface-based light-to-microwave transmitter for hybrid wireless communications

Supplementary movie 1

## Data Availability

The data that support the plots within this paper and other findings of this study are available from the corresponding author upon reasonable request.
