# Supplementary material for: A metasurface-based light-to-microwave transmitter for hybrid wireless communications
Source: Light Sci Appl. 2022 May 6;11:126. doi: 10.1038/s41377-022-00817-5 (PMC9072331; doi:10.1038/s41377-022-00817-5)
Supplement: Supplementary file 1 — Supplementary information for A metasurface-based light-to-microwave transmitter for hybrid wireless communications [file 41377_2022_817_MOESM1_ESM.docx]

**Supplementary information for**

**A metasurface-based light-to-microwave transmitter for hybrid wireless communications**

Xin Ge Zhang^1,#^, Ya Lun Sun^1,#^, Bingcheng Zhu^2,3,4#^, Wei Xiang Jiang^1,3,4^*, Qian Yu^1^, Han Wei Tian^1^, Cheng-Wei Qiu^5^, Zaichen Zhang^2,3,4^* and Tie Jun Cui^1^*

^1^ State Key Laboratory of Millimeter Waves, School of Information Science and Engineering, Southeast University, Nanjing 210096, China

^2^ National Mobile Communications Research Laboratory, School of Information Science and Engineering, Southeast University, Nanjing 210096, China

^3^ Frontiers Science Center for Mobile Information Communication and Security, Southeast University, Nanjing 210096, China

^4^ Purple Mountain Laboratories, Nanjing 211111, China

^5^ Department of Electrical and Computer Engineering, National University of Singapore, Singapore 117583, Singapore

These authors contributed equally: Xin Ge Zhang, Ya Lun Sun, Bingcheng Zhu.

E-mails: [wxjiang81@seu.edu.cn](mailto:wxjiang81@seu.edu.cn), zczhang@seu.edu.cn and [tjcui@seu.edu.cn](mailto:tjcui@seu.edu.cn)


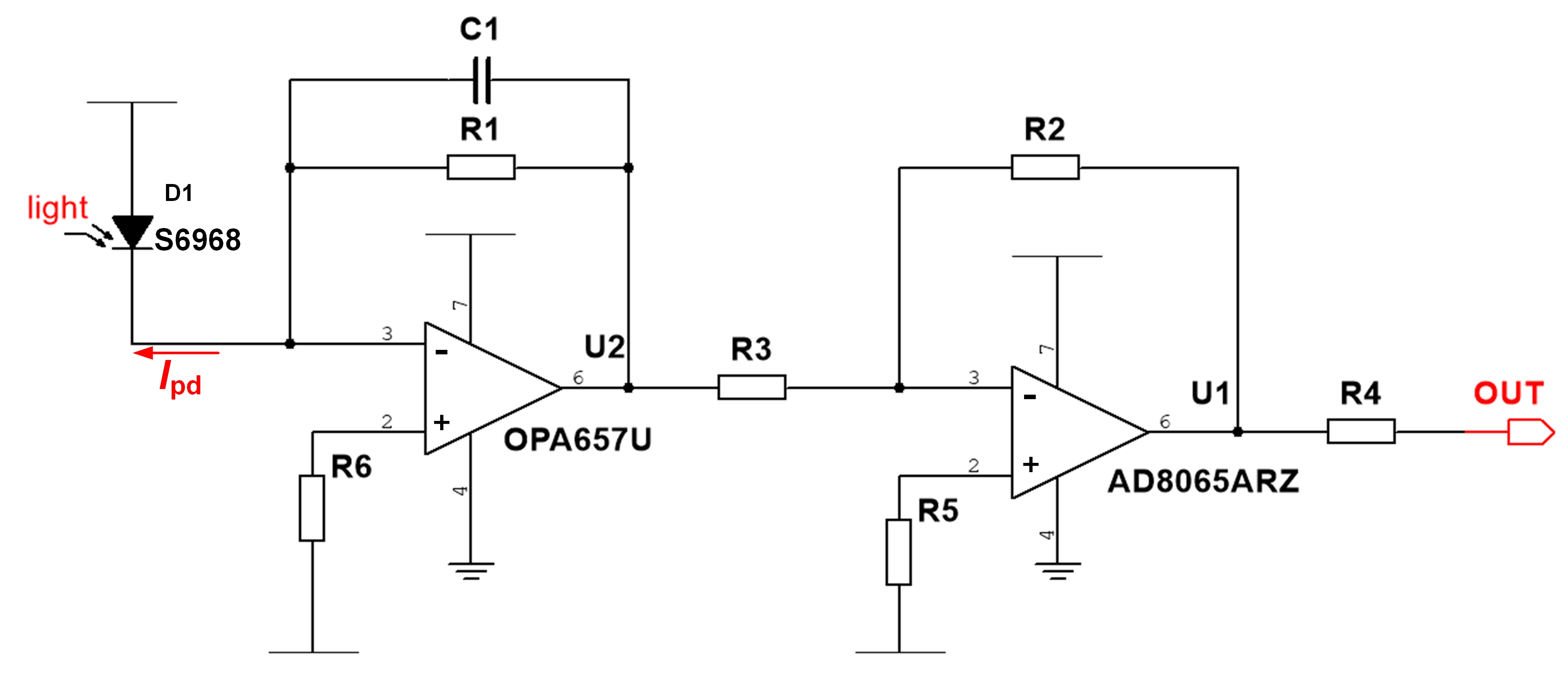


**Figure S1. Simplified diagram of the designed photoelectric detection circuit.** The photoelectric detection circuit has a high response speed and a high voltage output.


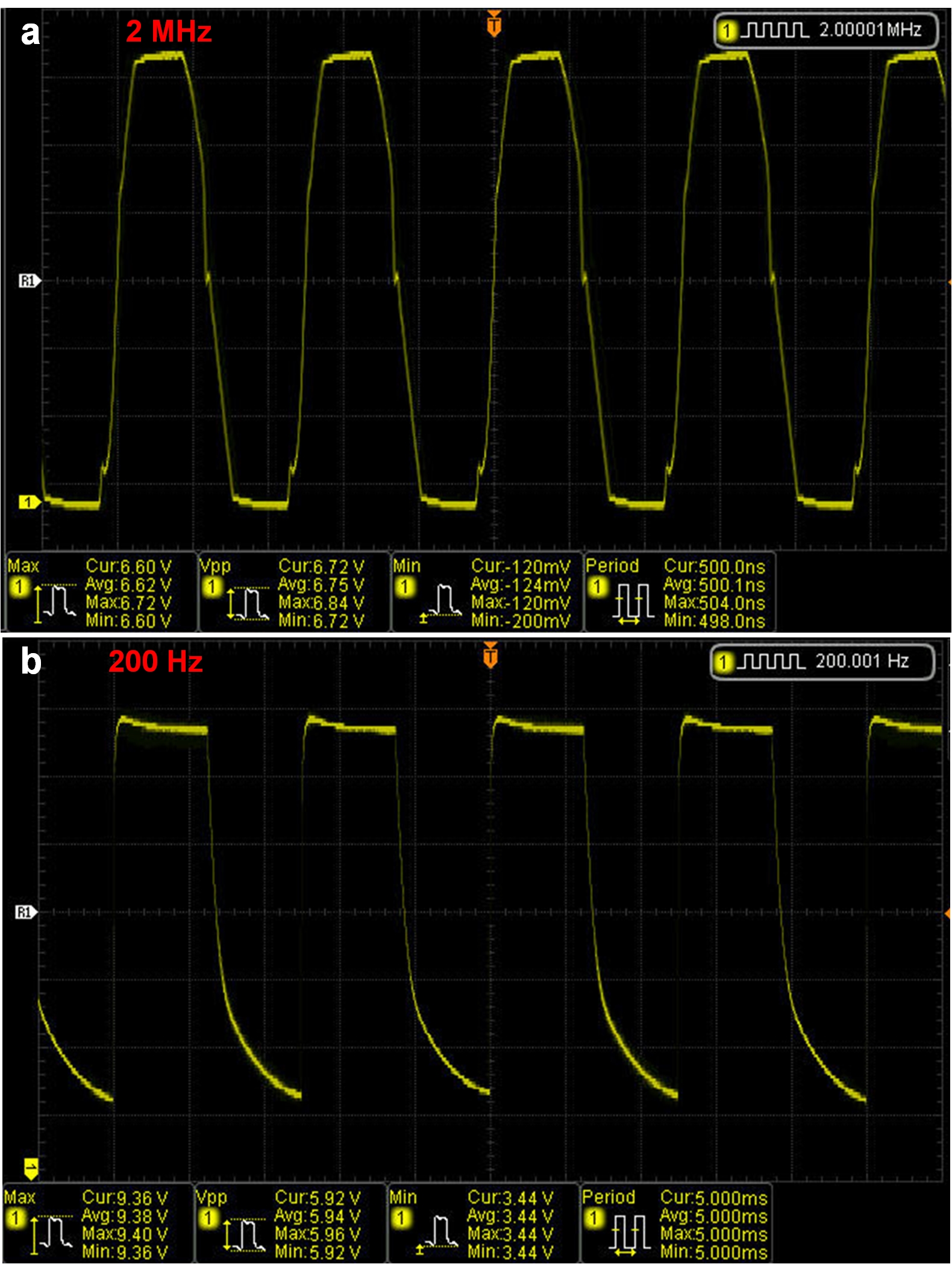


**Figure S2. Measured output voltage waveforms of the designed photoelectric detection circuit and the series photodiode array.** Under the illumination of time-varying light with the periodic square wave frequency, the switching frequency of the photoelectric detection circuit is able to reach 2 MHz (**a**), but that of the photodiode array is only about 200 Hz (**b**).

**Supplementary Note 1. Frequency manipulation based on hybrid time-varying metasurface**

When the time-varying metasurface is controlled by the up-modulation light waveform (light intensity increases with time, blue curve), it will blue-shift the incident frequency to a new frequency component upon reflection. In contrast, the time-varying metasurface is able to red-shift the frequency of incident microwaves for the down-modulation light waveform (light intensity decreases with time, red curve). The modulation speed of the time-varying metasurface mainly depends on the response time of the varactors. Here, the modulation frequency *f*_t_ of the metasurface is set as 100 kHz. The light waveform signals are generated by the designed optical transmitter (see **Materials and methods** in the main text). As examples, we present the measured normalized spectral power distributions of the reflected microwaves at three different frequencies of 3.7, 5.2, and 6.0 GHz under the normal incidence of *x*-polarized waves, as shown in **Fig. S3a-c**. From **Fig. S3a** we observe that at 3.7 GHz when the corresponding up- and down-modulation light signals are applied, the main frequency components of reflected waves are at 3.7001 and 3.6999 GHz, respectively, both producing an accurate 100 kHz artificial frequency shift. At 5.2 and 6.0 GHz, the time-varying metasurface can also generate 100 kHz blue- and red-shifts under the corresponding light modulation signals, as shown respectively in **Fig. S3b,c**, which further verifies the broadband characteristic of the metasurface. For the three frequencies, the measured energies of the generated blue- and red-shifted components are all 10 dB larger than those of the fundamental frequencies, showing a high frequency conversion efficiency.


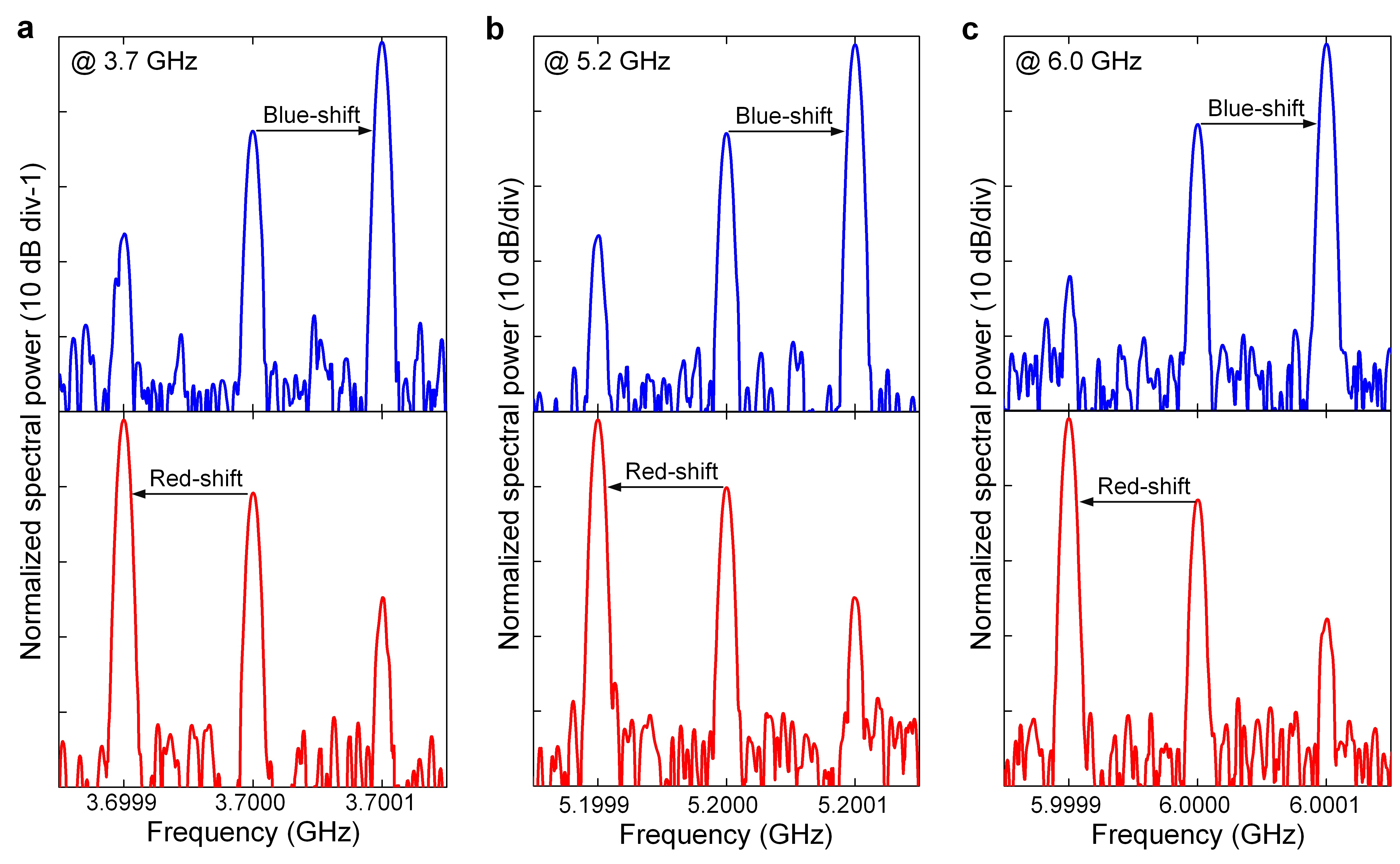


**Figure S3. Measured normalized spectral power distributions of the reflected microwaves at three different frequencies. a-c**, Measured results of the optically programmed time-varying metasurface at 3.7, 5.2, and 6.0 GHz, respectively.

**Supplementary Note 2. Dispersion analysis of the metasurface**

We observe a strong nonlinear relationship between the measured reflection phase and the light intensity. Such a nonlinear effect is mainly caused by the inherent nonlinear response of the varactor. In the region where the phase changes sharply, when the operating frequency is slightly changed, the reflection phase of the metasurface will be significantly different at a fixed light intensity. This indicates that the designed metasurface has a strong dispersion. We will utilize the dispersion feature to realize dual-channel signal conversion, as discussed in the main text.

**Supplementary Note 3. Characteristic of light waveform**

The control waveform of light signal is designed to make the reflection phase change linearly with time. Because each reflection phase curve has a sharply changed region, the corresponding light waveform has a gentle area where the light intensity is almost constant. This gently varying waveform is the key to ensure that the reflection phase changes linearly in one modulation period. As operating frequency increases, the light intensity in the gently varying region also rises.

**Supplementary Note 4. Video transmission process**

The two videos are received at the same time and played in real time. When the light source is turned off, there is no data transmission and only noise exists. When we turn on the light source, the two videos can be recovered immediately. In addition, when we use a metal plate to cover the receiving horn antenna 2, the video 2 recovered from the corresponding signal becomes noise. After removing the obstruction, the video displays again. During this process, video 1 remains displayed. The transmission rate of the hybrid system is 100 kbps, corresponding to the 100 kHz modulation frequency of light waveform. The transmission rate of the hybrid communication system mainly depends on the response speeds of the designed photoelectric detection circuit and the varactor-based programmable metasurface as well as the used modulation mode. Although the switching frequency of the photoelectric detection circuit can reach 2 MHz, the programmable metasurface has a slower switching speed due to a lot of parallel varactors loading. In addition, to achieve the dual-channel data transmission, we use the basic BFSK modulation format to implement the FDM scheme. To improve the communication rate of the hybrid system, an effective solution is to develop high-order modulation formats, such as phase shift keying (PSK) and quadrature amplitude modulation (QAM) on the programmable metasurface.


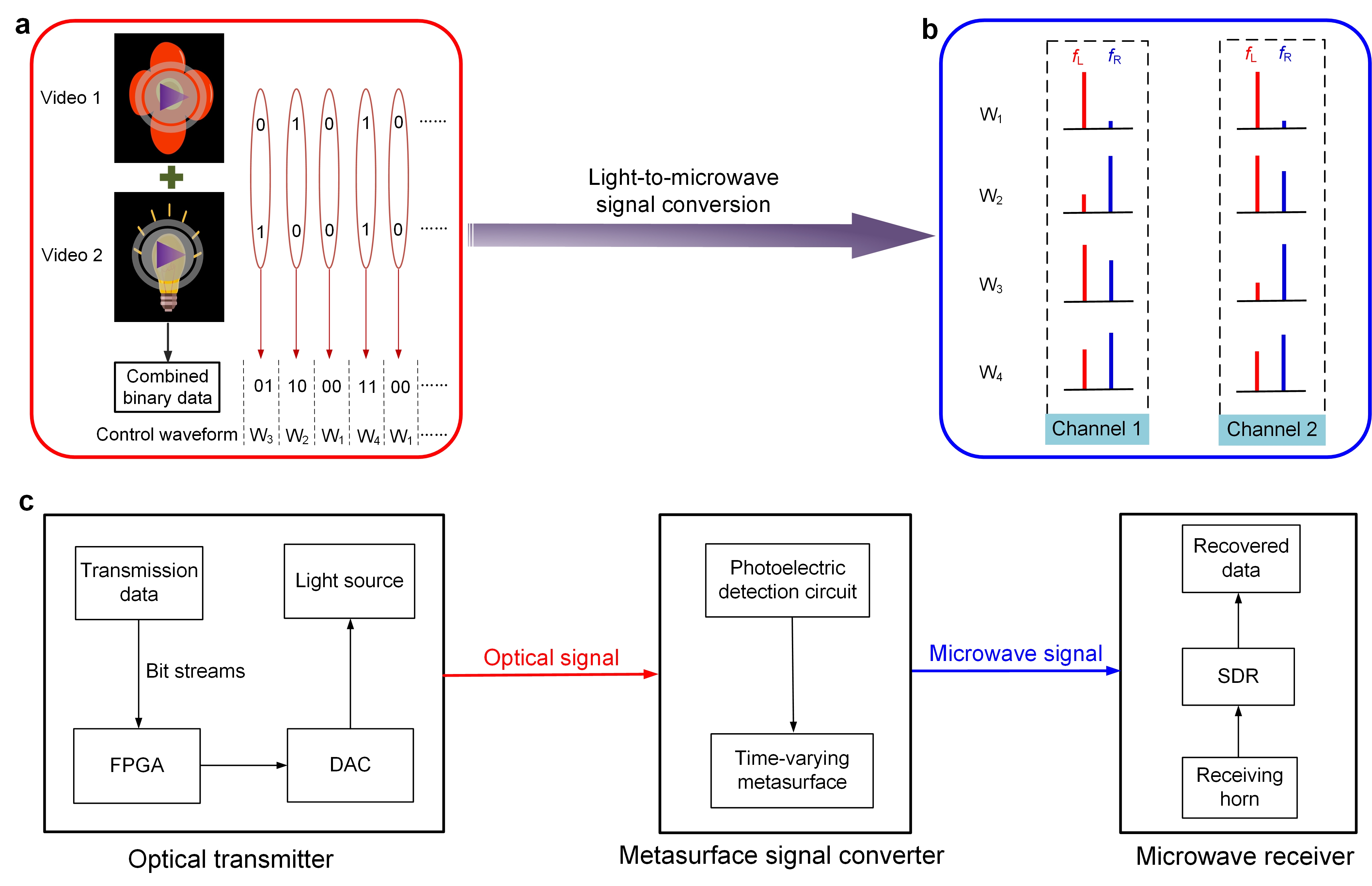


**Figure S4. Workflow of the dual-channel hybrid communication system based on the metasurface-enabled light-to-microwave signal converter. a**, Optical signal generation, in which video 1 and video 2 are encoded onto the four different sets of light waveforms. **b**, Four sets of spectral distributions used to generate two BFSK signals. **c**, Block diagram of the complete workflow of the hybrid communication system.
